# Efficacy and safety of bispecific antibodies therapy for relapsed or refractory multiple myeloma: a systematic review and meta-analysis of prospective clinical trials

**DOI:** 10.3389/fimmu.2024.1348955

**Published:** 2024-02-28

**Authors:** Xin Wang, Ailin Zhao, Jinbing Zhu, Ting Niu

**Affiliations:** Department of Hematology, West China Hospital, Sichuan University, Chengdu, China

**Keywords:** bispecific antibodies, immunotherapy, multiple myeloma, efficacy, safety

## Abstract

**Objective:**

Bispecific antibody (BsAbs) therapy represents a promising immunotherapeutic approach with manageable toxicity and noteworthy preliminary efficacy in treating patients with relapsed or refractory multiple myeloma (RRMM). The objective of this systematic review and meta-analysis was to compare the efficacy and safety of B-cell maturation antigen (BCMA)-targeted BsAbs and non-BCMA-targeted BsAbs in the treatment of RRMM patients.

**Methods:**

PubMed/MEDLINE, Web of Science, EMBASE, Cochrane Library and meeting libraries were searched from inception to August 16th, 2023. The efficacy evaluation included the complete objective response rate (ORR), complete response (CR) rate, stringent CR (sCR) rate, partial response (PR) rate, and very good PR (VGPR) rate. The efficacy evaluation included any grade adverse events (AEs) and grade ≥ 3 AEs.

**Results:**

Fourteen studies with a total of 1473 RRMM patients were included. The pooled ORR of the entire cohort was 61%. The non-BCMA-targeted BsAbs group displayed a higher ORR than the BCMA-targeted BsAbs group (74% *vs*. 54%, *P* < 0.01). In terms of hematological AEs, BCMA-targeted BsAbs therapy exhibited higher risks of neutropenia (any grade: 48% *vs*. 18%, *P* < 0.01; grade ≥ 3: 43% *vs*. 15%, *P* < 0.01) and lymphopenia (any grade: 37% *vs*. 8%, *P* < 0.01; grade ≥ 3: 31% *vs*. 8%, *P* = 0.07). Regarding non-hematological AEs, there were no significant differences in the risks of cytokine release syndrome (CRS, any grade: 64% *vs*. 66%, *P* = 0.84; grade ≥ 3: 1% *vs*. 1%, *P* = 0.36) and infections (any grade: 47% *vs*. 49%, *P* = 0.86; grade ≥ 3: 24% *vs*. 20%, *P* = 0.06) between the two groups. However, non-BCMA-targeted BsAbs therapy was associated with a higher risk of immune effector cell-associated neurotoxicity syndrome (ICANS, any grade: 11% *vs*. 2%, *P* < 0.01) and lower risks of fatigue (any grade: 14% *vs*. 30%, *P* < 0.01) and pyrexia (any grade: 14% *vs*. 29%, *P* < 0.01).

**Conclusion:**

This analysis suggest that non-BCMA-targeted BsAbs therapy may offer a more favorable treatment response and tolerability, while BCMA-targeted BsAbs therapy may be associated with diminished neurotoxic effects.

**Systematic Review Registration:**

https://www.crd.york.ac.uk/PROSPERO/, identifier CRD42018090768.

## Introduction

Multiple myeloma (MM) is characterized by uncontrolled proliferation of clonal plasma cells, leading to myeloma-defining events (1, 2). Despite advancements in treatment, a substantial number of patients with MM experience relapse and develop resistance to conventional therapies, rendering the disease largely incurable (3, 4). With a deepening understanding of disease biology, innovative therapeutic approaches continue to emerge.

Bispecific antibodies (BsAbs) therapy is a novel approach that has shown potential in early phase trials for the treatment of relapsed or refractory MM (RRMM) (5, 6). BsAbs bind a target on both tumor cells and effector T-cells, which results in T-cell activation and thereby tumor cell apoptosis (7, 8). There are several BsAb formats, most of BsAbs used in MM target B-cell maturation antigen (BCMA), whereas others target non-BCMA antigens, including G protein-coupled receptor, class C group 5 member D (GPRC5D), Fc receptor-like protein 5 (FcRH5), and CD38 (9, 10).

In several ongoing trials, however, severe adverse events (AEs) have been observed with BsAbs, such as cytopenias, infections, cytokine release syndrome (CRS) and neurotoxicity (11, 12). To enhance clinical understanding of these therapies, we summarized pivotal data, including benefits and risks between BCMA-targeted and non-BCMA-targeted BsAbs in this study.

## Methods

### Search strategy and selection criteria

Relevant clinical studies were identified by a systematic search of PubMed/MEDLINE, Web of Science, EMBASE, and Cochrane Library using the following MeSH Terms: “multiple myeloma”, “antibodies, bispecific” and corresponding Entry Terms. Additional records were retrieved by screening published conference abstracts of the American Society of Clinical Oncology (ASCO), American Society of Hematology (ASH), and European Hematology Association (EHA). Only prospective clinical trials registered on Clinicaltrials.gov (NCT-number), either as full articles or as abstracts during the annual meetings of ASCO, ASH, or EHA, were taken into consideration. The search included only texts published before August 16th, 2023. There were no restrictions on language, follow-up or study size. The analysis was registered in PROSPERO (CRD42018090768).

### Exclusion criteria

The exclusion criteria were as follows: 1) insufficient data on efficacy or safety; 2) reviews, case reports, news, editorials, and meta-analyses; and 3) terminated/suspended due to the sponsor business decision.

### Data extraction and quality assessment

Two authors independently screened the literature and collected the data, and any difference was settled by the third author. BsAbs were categorized based on their targets as BCMA *vs*. non-BCMA. The extracted data were sorted into a designed spreadsheet that mainly included the first author, ClinicalTrials.gov number, phase, number of patients, ages, treatment, target, prior exposure to anti-BCMA treatment, prior line of treatment (LOT), any grade AEs, any grade CRS, ORR, median progression-free survival (mPFS), and median duration of response (mDOR). Information about BsAbs treatment was extracted. To avoid duplicate data, only the most recent records were included. For the included studies, the quality was estimated by the modified methodological index for nonrandomized studies (MINORS) (13, 14).

### Statistical analysis

Statistical analysis of the data was performed using R 4.3.1 software. All statistical tests were two-sided, and *P* < 0.05 was considered statistically significant. The I² statistic test was applied to appraise the heterogeneity among studies. A fixed-effects model was employed if I^2^ ≤ 50%, while a random-effects model was utilized if I^2^ > 50%. Effects were expressed as pooled event rates with 95% confidence intervals (CI).

## Results

### Study characteristics

**Figure 1:**
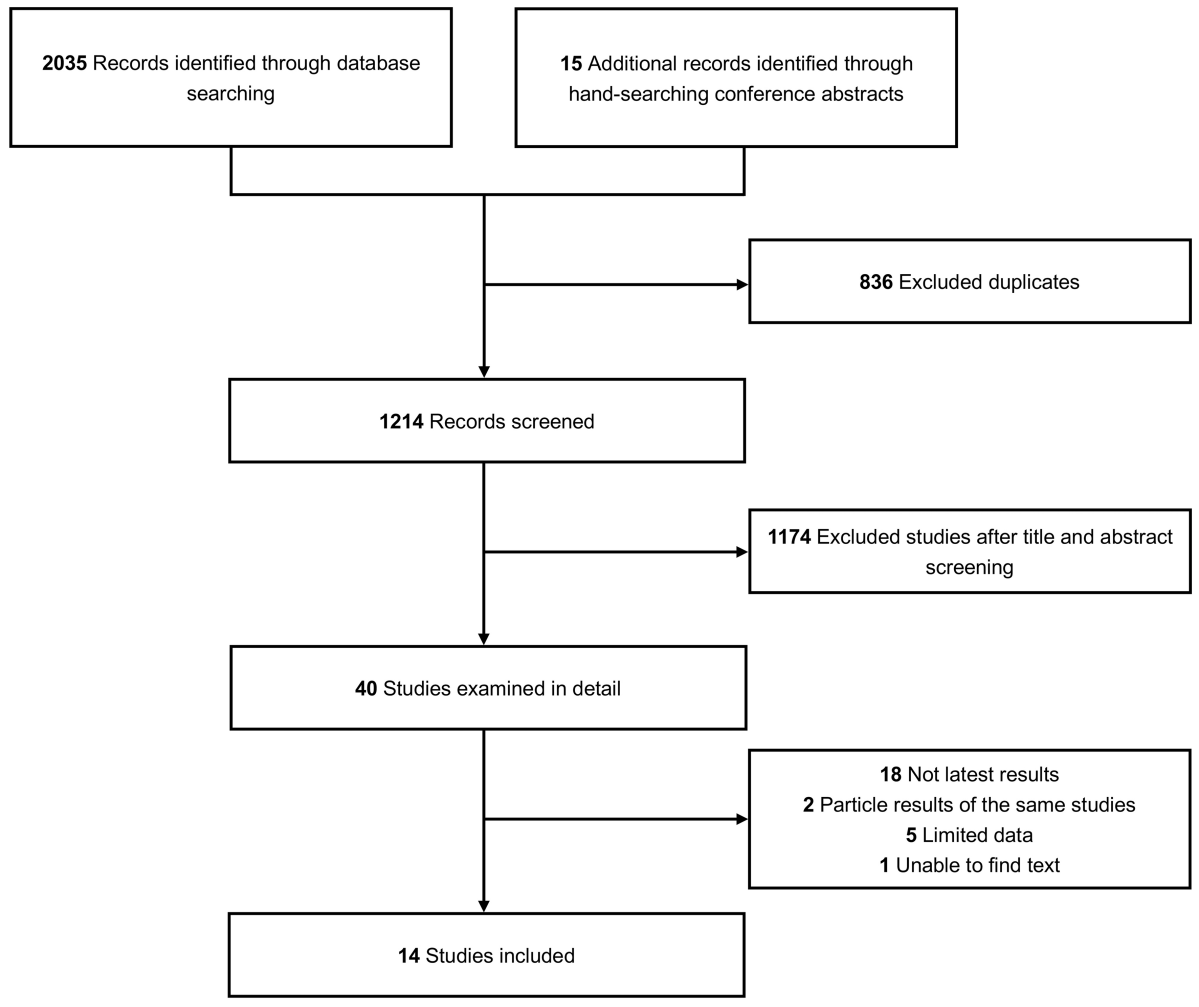
The flow chart.

A total of 2040 studies describing BsAbs for RRMM were included, with an additional 15 pertinent studies identified from conference abstracts. Fourteen qualified studies were identified in the final analysis (15–28). The complete screening process is illustrated in **Figure 1**. The study included 1473 patients in total—829 patients underwent BCMA-targeted BsAbs treatment, and 644 patients underwent non-BCMA-targeted BsAbs treatment. All data were derived from phase 1 and 2 clinical trials. The median prior LOT of all patients ranged from 4-6. The drugs included in this analysis were teclistamab, F182112, linvoseltamab (50mg), linvoseltamab (200mg), elranatamab, TNB-383B (40mg), TNB-383B (60mg), alnuctamab (SC), alnuctamab (IV), WVT078, talquetamab (40mg SC), talquetamab (80mg SC), talquetamab (IV), RG6234 (SC), RG6234 (IV), cevostamab, ISB-1342. Further details of the included studies are shown in **Table 1**.

**Table 1:** The characteristics of the included studies.

Study	Trial #	Phase	Number of pts	Age (years), median (range)	Treatment	Usage	Target	Prior exposure to anti-BCMA treatment	Prior LOT	Any grade AEs (%)	Any grade CRS (%)	ORR (%)	mPFS (m)	mDOR (m)
Moreau et al., 2022 (28)	NCT03145181 NCT04557098	1-2	165	64 (33-84)	Teclistamab	SC 1.5 mg/kg	BCMA×CD3	Not allowed (cohort A)	5 (2-11)	100	72	63	11.3	18.4
Touzeau et al., 2022 (20)	NCT03145181 NCT04557098	1-2	38	63.5 (32-82)	Teclistamab	SC 1.5 mg/kg	BCMA×CD3	The enrolled pts had previously received anti-BCMA therapies (cohort C)	6 (3-14)	–	63	40	–	NR
Sun et al., 2023 (26)	NCT04984434	1	16	64 (52-74)	F182112	IV 0.01-20 µg/kg	BCMA×CD3	–	–	–	81	44	–	–
Lee et al., 2023 (23)	NCT03761108	2	252	66 (37-90)	Linvoseltamab	IV (a) 50 mg (b) 200 mg	BCMA×CD3	–	5 (1-16)	(a) 95 (b) 100	(a) 37 (b) 53	(a) 64 (b) 50	–	NR
Lesokhin et al., 2023 (18)	NCT04649359	2	123	68 (36-89)	Elranatamab	SC 12, 32 and 76 mg	BCMA×CD3	Not allowed (cohort A)	5 (2-22)	100	58	61	NR	NR
Voorhees et al., 2022 (15)	NCT03933735	1	66	(c) 64 (56-76) (d) 68 (35-92)	TNB-383B	IV (c) 40 mg (d) 60 mg	BCMA×CD3	Not allowed	(c) 4 (3-10) (d) 5 (3-12)	(c) 100 (d) 98	(c) 83 (d) 72	(c) 83 (d) 60	NR	NR
Wong et al., 2022 (16)	NCT03486067	1	117	(e) - (f) 64 (-)	Alnuctamab	(e) IV 0.15-10 mg (f) SC 3-60 mg	BCMA×CD3	Not allowed	–	(e) - (f) 89	(e) - (f) 53	(e) 39 (f) 51	–	(e) 34.09 (f) -
Costa et al., 2019 (21)	NCT03486067	1	19	64 (51-78)	Alnuctamab	IV 0.15-10 mg	BCMA×CD3	Not allowed	6 (3-12)	–	90	–	–	–
Raab et al., 2023 (25)	NCT04123418	1	33	64 (50-75)	WVT078	IV 3, 6, 12, 24, 48, 64, 96, 192 and 250 µg/kg	BCMA×CD3	6.1% pts had previously received anti-BCMA therapies	5 (2-13)	85	61	39	–	–
Schinke et al., 2023 (24)	NCT03399799 NCT04634552	2	339	–	Talquetamab	SC (g,i) 0.4 mg/kg (h,i) 0.8 mg/kg	GPRC5D×CD3	–	–	–	(g) 79 (h) 75 (i) 77	(g) 74 (h) 73 (i) 63	(g) 7.5 (h) 11.9 (i) 5.1	–
Carlo-Stella et al., 2022 (27)	NCT04557150	1	105	(j) 62 (27-78) (k) 62 (46-79)	RG6234	(j) IV 6-10000 µg (k) SC 30-7200 µg	GPRC5D×CD3	20% pts had previously received anti-BCMA therapies	(j) 5 (2-15) (k) 4 (2-14)	–	(j) 82 (k) 78	(j) 71 (k) 60	–	–
Lesokhin et al., 2022 (19)	NCT03275103	1	16	66.5 (45-80)	Cevostamab	IV 40-160 mg	FcRH5×CD3	31.25% pts had previously received anti-BCMA therapies	6 (2-11)	–	–	100	–	–
Trudel et al., 2021 (17)	NCT03275103	1	160	64 (33-82)	Cevostamab	IV 0.05-3.6 and 0.15-198 mg, or 0.3-1.2, 3.6 and 60-160 mg	FcRH5×CD3	33.8% pts had previously received anti-BCMA therapies	6 (2-18)	99	80	–	–	–
Mohan et al., 2022 (22)	NCT03309111	1	24	67 (54-76)	ISB-1342	IV 0.2/0.3-1.0/4.0 mg/kg	CD38×CD3	33% pts had previously received anti-BCMA therapies	6 (1-10)	92	17	–	–	–

AE, adverse event; BCMA, B-cell maturation antigen; BsAbs, bispecific antibodies; pts, patients; mPFS, median duration of progression-free survival; mDOR, median duration of response; NR, not reached; GPRC5D, G-protein coupled receptor family C group 5 member D; FcRH5, Fc Receptor-Like 5; IV, intravenous; SC, subcutaneous.

1) The two dose cohorts in the trial of linvoseltamab were analyzed separately: (a) 50 mg and (b) 200 mg. 2) The two dose cohorts in the trial of TNB-383B were analyzed separately: (c) 40 mg and (d) 60 mg. 3) The two usage cohorts in the trial of alnuctamab were separately analyzed separately: (e) intravenous and (f) subcutaneous administration. 4) The different doses and groups of patient cohorts in the trial of talquetamab were analyzed separately: (g) 0.4 mg/kg; previous treatment with T-cell redirection therapy was not allowed, (h) 0.8 mg/kg; previous treatment with a T-cell redirection therapy was not allowed, and (i) 0.4 mg/kg or 0.8 mg/kg; patients enrolled had prior exposure to T-cell redirection therapy. 5) The two usage cohorts in the trial of RG6234 were analyzed separately: (j) intravenous and (k) subcutaneous administration. 6) Trial # = study registration number in ClinicalTrials.gov (NCT#).

### Efficacy

To evaluate the efficacy of BCMA-targeted BsAbs and non-BCMA-targeted BsAbs therapies for RRMM, we synthesized data on ORR, complete response (CR), stringent CR (sCR), partial response (PR), very good PR (VGPR) and ≥ VGPR.

Eight and six studies described ORR in the BCMA-targeted BsAbs group and non-BCMA-targeted BsAbs group, respectively. The pooled ORR of the entire cohort was 61% (95%CI: 54%-69%). Notably, the non-BCMA-targeted BsAbs group displayed a higher ORR than the BCMA-targeted BsAbs group (74% *vs*. 54%, *P* < 0.01).

**Figure 2:**
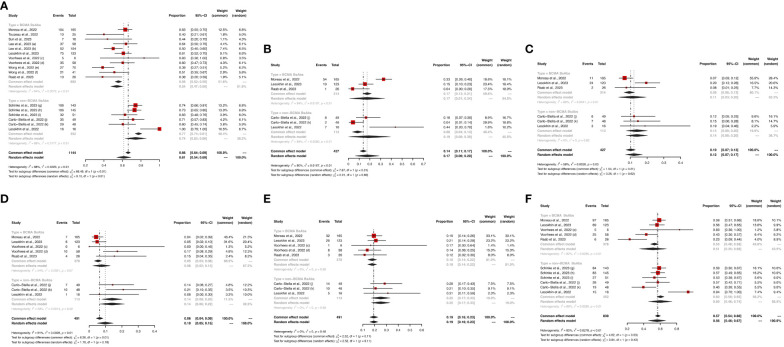
The pooled **(A)** ORR, **(B)** sCR, **(C)** CR, **(D)** PR, **(E)** VGPR and **(F)** ≥VGPR in patients treated with BsAbs. 1) The two dose cohorts in the trial of linvoseltamab were analyzed separately: **(a)** 50 mg and **(b)** 200 mg. 2) The two dose cohorts in the trial of TNB-383B were analyzed separately: **(c)** 40 mg and **(d)** 60 mg. 3) The two usage cohorts in the trial of alnuctamab were separately analyzed separately: **(e)** intravenous and **(f)** subcutaneous administration. 4) The different doses and groups of patient cohorts in the trial of talquetamab were analyzed separately: **(g)** 0.4 mg/kg; previous treatment with T-cell redirection therapy was not allowed, **(h)** 0.8 mg/kg; previous treatment with a T-cell redirection therapy was not allowed, and **(i)** 0.4 mg/kg or 0.8 mg/kg; patients enrolled had prior exposure to T-cell redirection therapy. 5) The two usage cohorts in the trial of RG6234 were analyzed separately: **(j)** intravenous and **(k)** subcutaneous administration.

For the BCMA-targeted BsAbs group, the pooled sCR, CR, PR, VGPR and ≥ VGPR and were 17% (95%CI: 1%-34%), 11% (95%CI: 3%-20%), 8% (95%CI: 2%-13%), 18% (95%CI: 14%-22%), and 51% (95%CI: 35%-68%), respectively. For the non-BCMA-targeted BsAbs group, the pooled sCR, CR, PR, VGPR and ≥ VGPR were 19% (95%CI: 0%-39%), 14% (95%CI: 8%-20%), 14% (95%CI: 8%-20%), 25% (95%CI: 17%, 33%) and 60% (95%CI: 46%-74%), respectively. The specific results are shown in **Figure 2**.

### Safety

In terms of hematological AEs, BCMA-targeted BsAbs therapy exhibited higher risks of neutropenia (any grade: 48% *vs*. 18%, *P* < 0.01; grade ≥ 3: 43% *vs*. 15%, *P* < 0.01) and lymphopenia (any grade: 37% *vs*. 8%, *P* < 0.01; grade ≥ 3: 31% *vs*. 8%, *P* = 0.07). No significant differences were observed in the risks of anemia (any grade: 38% *vs*. 30%, *P* = 0.14; grade ≥ 3: 23% *vs*. 11%, *P* = 0.05) and thrombocytopenia (any grade: 31% *vs*. 17%, *P* = 0.11; grade ≥ 3: 17% *vs*. 10%, *P* = 0.27) between the two groups.

Regarding non-hematological AEs, there were no significant differences in the risks of CRS (any grade: 64% *vs*. 66%, *P* = 0.84; grade ≥ 3: 1% *vs*. 1%, *P* = 0.36) and infections (any grade: 47% *vs*. 49%, *P* = 0.86; grade ≥ 3: 24% *vs*. 20%, *P* = 0.06) between the two groups. However, non-BCMA-targeted BsAbs therapy was associated with a higher risk of immune effector cell-associated neurotoxicity syndrome (ICANS, any grade: 11% *vs*. 2%, *P* < 0.01) and lower risks of fatigue (any grade: 14% *vs*. 30%, *P* < 0.01) and pyrexia (any grade: 14% *vs*. 29%, *P* < 0.01). There were no significant differences in the risks of any grade AEs (100% *vs*. 99%, *P* = 0.50) or grade ≥ 3 AEs (73% *vs*. 50%, *P* = 0.05) between the two groups.

**Figure 3:**
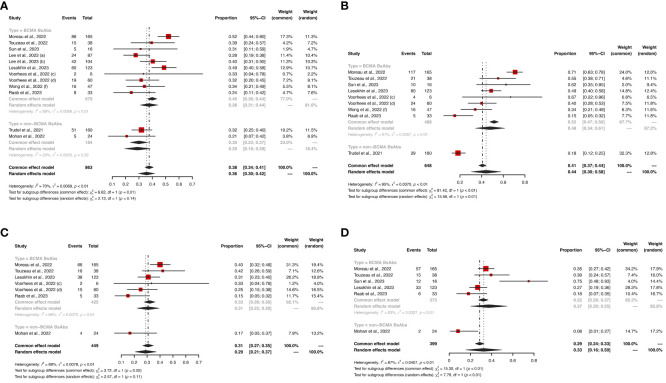
The pooled risks of any grade **(A)** anemia, **(B)** neutropenia, **(C)** thrombocytopenia and **(D)** lymphopenia in patients treated with BsAbs. 1) The two dose cohorts in the trial of linvoseltamab were analyzed separately: **(a)** 50 mg and **(b)** 200 mg. 2) The two dose cohorts in the trial of TNB-383B were analyzed separately: **(c)** 40 mg and **(d)** 60 mg. 3) The **(f)** subcutaneous administration cohort in the trial of alnuctamab was analyzed separately.

**Figure 4:**
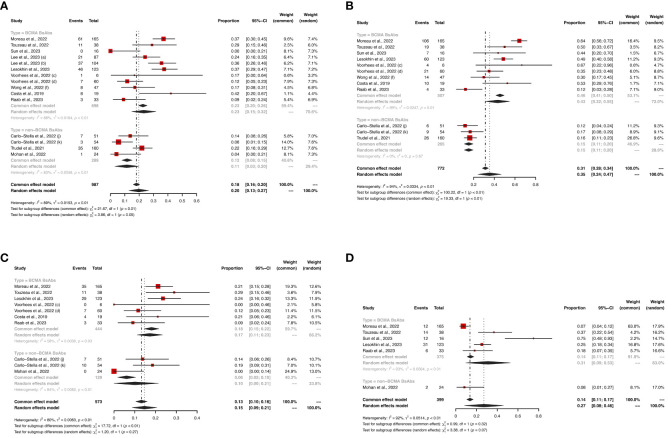
The pooled risk of grade ≥ 3 **(A)** anemia, **(B)** neutropenia, **(C)** thrombocytopenia and **(D)** lymphopenia in patients treated with BsAbs. 1) The two dose cohorts in the trial of linvoseltamab were analyzed separately: **(a)** 50 mg and **(b)** 200 mg. 2) The two dose cohorts in the trial of TNB-383B were analyzed separately: **(c)** 40 mg and **(d)** 60 mg. 3) The **(f)** subcutaneous administration cohort in the trial of alnuctamab was analyzed separately. 4) The two usage cohorts in the trial of RG6234 were analyzed separately: **(j)** intravenous and **(k)** subcutaneous administration.

**Figure 5:**
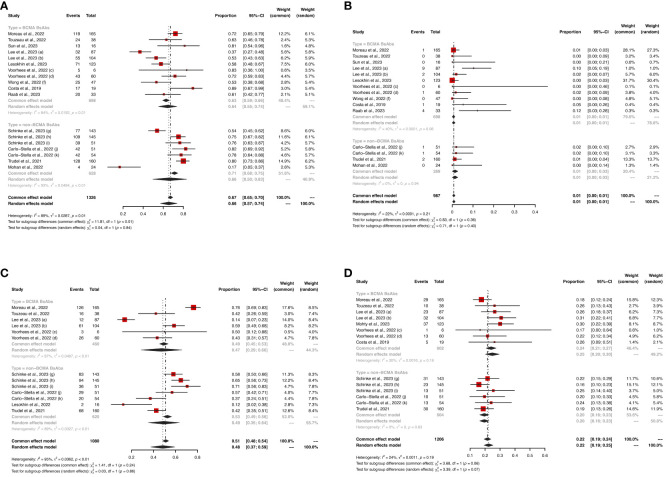
The pooled risks of **(A)** any grade CRS, **(B)** grade ≥ 3 CRS, **(C)** any grade infections, and **(D)** grade ≥ 3 infections in patients treated with BsAbs. 1) The two dose cohorts in the trial of linvoseltamab were analyzed separately: **(a)** 50 mg and **(b)** 200 mg. 2) The two dose cohorts in the trial of TNB-383B were analyzed separately: **(c)** 40 mg and **(d)** 60 mg. 3) The **(f)** subcutaneous administration cohort in the trial of alnuctamab was analyzed separately. 4) The different doses and groups of patient cohorts in the trial of talquetamab were analyzed separately: **(g)** 0.4 mg/kg; previous treatment with T-cell redirection therapy was not allowed, **(h)** 0.8 mg/kg; previous treatment with a T-cell redirection therapy was not allowed, and **(i)** 0.4 mg/kg or 0.8 mg/kg; patients enrolled had prior exposure to T-cell redirection therapy. 5) The two usage cohorts in the trial of RG6234 were analyzed separately: **(j)** intravenous and **(k)** subcutaneous administration.

Hematological AEs for BCMA-targeted BsAbs *vs*. non-BCMA-targeted BsAbs therapies are shown in **Figures 3**, **4**, and non-hematological AEs are shown in **Figure 5** and **Table 2**.

**Table 2:** The incidence of adverse events for patients with RRMM.

Event	Treatment	Any grade					Grade ≥ 3				
		Included study	Events	Total	Pooled rate [95% Cl]	*P*	Included study	Events	Total	Pooled rate [95% Cl]	*P*
Any AE	BCMA BsAbs	6	610	625	1.00 [0.99; 1.00]	0.50	7	494	644	0.73 [0.64; 0.82]	0.05
	non-BCMA BsAbs	2	181	184	0.99 [0.98; 1.00]		2	103	184	0.50 [0.30; 0.71]	
Fatigue	BCMA BsAbs	4	158	512	0.30 [0.24; 0.36]	**< 0.01**	3	8	321	0.02 [0.01; 0.04]	0.65
	non-BCMA BsAbs	2	28	184	0.15 [0.10; 0.20]		1	3	184	0.02 [0.00; 0.04]	
Infusion-reactions	BCMA BsAbs	1	6	33	0.18 [0.07; 0.35]	0.49	1	1	33	0.03 [0.00; 0.16]	0.68
	non-BCMA BsAbs	2	38	184	0.28 [0.04; 0.51]		2	4	184	0.07 [0.00; 0.23]	
Diarrhea	BCMA BsAbs	3	104	321	0.29 [0.14; 0.44]	0.40	3	8	321	0.02 [0.00; 0.04]	0.17
	non-BCMA BsAbs	2	45	184	0.21 [0.07; 0.34]		2	1	184	0.01 [0.00; 0.02]	
Pyrexia	BCMA BsAbs	3	95	321	0.29 [0.24; 0.34]	**< 0.01**	3	7	321	0.01 [0.00; 0.02]	0.14
	non-BCMA BsAbs	2	27	184	0.14 [0.09; 0.19]		2	0	184	0.00 [0.00; 0.01]	
ICANS	BCMA BsAbs	3	6	245	0.02 [0.00; 0.04]	**< 0.01**	–	–	–	–	–
	non-BCMA BsAbs	1	32	288	0.11 [0.07; 0.15]		–	–	–	–	
Nausea	BCMA BsAbs	3	82	321	0.23 [0.14; 0.32]	0.84	3	1	321	0.00 [0.00; 0.01]	0.65
	non-BCMA BsAbs	1	35	160	0.22 [0.16; 0.29]		1	0	160	0.00 [0.00; 0.01]	

RRMM, relapsed/refractory multiple myeloma; AE, adverse event; BCMA, B-cell maturation antigen; BsAbs, bispecific antibodies; ICANS, immune effector cell-associated neurotoxicity syndrome. All statistically significant values are recorded in bold.

### Quality assessment

**Supplementary Table S1** summarizes the quality assessment for the 14 included studies based on the modified MINORS criteria. Scores ranged from 9 to 15, with a median value of 11. Common weaknesses included an incomplete statement of outcome evaluation bias, incomplete reporting of completeness of follow-up, and inadequate follow-up period. Overall, the quality of the enrolled studies was deemed acceptable.

## Discussion

Patients with RRMM often encounter challenges associated with multiple lines of treatment and poor clinical outcomes, highlighting the imperative to investigate novel and effective therapeutic alternatives (29). BsAb recognize two epitopes or antigens and are among the most promising immunotherapeutic drugs for RRMM today (30, 31). In this first, large-scale systematic review and meta-analysis, we quantified the reported efficacy and safety of BsAbs in RRMM.

In this analysis, the pooled ORR for the entire cohort was 61%. Monotherapy trials in this population that led to the U.S. Food and Drug Administration (FDA) approval, including teclistamab, elranatamab and talquetamab, achieved 40% to 74% ORR and 5.1 month to NR mPFS (18, 24, 28, 32, 33). Importantly, these trials were at different stages of maturity or recruited different groups of patients, and therefore had variable response rates. In the long-term follow-up from the MajesTEC-1 study, the ORRs were 63% and 40% for cohort A (previous treatment with a BCMA-targeted therapy was not allowed) and cohort C (patients enrolled had prior exposure to anti-BCMA treatment), respectively (20, 28). Similarly, in the pivotal cohorts of the MonumenTAL-1 study, ORRs were consistent across subgroups (74% and 73%), but in the prior T-cell redirection cohort, the ORR was as low as 63% (24). Future recommendations may need to take into account these variables.

Our findings highlight that non-BCMA-targeted BsAbs treatment is associated with a significantly improved ORR for patients with RRMM. Notably, except for one study, patients enrolled in other non-BCMA-targeted BsAbs studies previously received anti-BCMA treatment, accounting for 20% to 33.8% (17, 19, 22, 27). In a phase 1 study of RRMM treated with 1-year, fixed-duration cevostamab, all four patients who were refractory to anti-BCMA treatment achieved a response (19). The non-BCMA-targeted BsAbs in this analysis included GPRC5D, FcRH5 and CD38. BCMA, or CD269, is ubiquitously present on the surface of plasma cells including MM cells (34, 35). Whereas GPRC5D, FcRH5 are preferentially expressed on multiple myeloma cells (36). Patients with RRMM who have received prior anti-BCMA therapies may also benefit from non-BCMA-targeted BsAbs therapy.

Among BCMA-targeted BsAbs, Moreau showed that the median PFS and duration of response (DOR) for teclistamab were 11.3 and 18.4 months, respectively (27); Lesokhin reported that fifteen-month DOR and PFS rates for elranatamab were 71.5% and 50.9%, respectively (17); Wong revealed that the median DOR in patients with IV Alnuctamab was 146.1 weeks (15). These studies suggested that BCMA-targeted BsAbs exhibited deep and durable responses in patients with RRMM. When it comes to non-BCMA-targeted BsAbs, talquetamab displayed varied PFS across different patient populations and dosage groups, with median PFS observed at 7.5, 11.9, and 5.1 months in the 0.4mg/kg QW, 0.8mg/kg Q2W, and prior T-cell redirection cohorts, respectively (23). It is noteworthy that the current data on PFS and DOR is limited, necessitating longer-term follow-ups to provide extensive information.

Common AEs of BsAbs therapy included CRS, infections and neutropenia. Cytopenias were mainly high-grade, which may lead to an increased risk of serious opportunistic infections, while CRS events were almost limited to low-grade. Compared with subcutaneous (SC) administration, intravenous (IV) administration was related to a higher incidence of CRS. In the phase 1 study of alnuctamab (ALNUC), any grade of CRS was reported in 89.5% of patients treated with IV ALNUC and 63% of patients treated with SC ALNUC (16, 21). Low-grade CRS was generally be treated with antipyretics, analgesics and corticosteroids (17, 24, 27). In addition, tocilizumab was highly effective and widely used for treating CRS and ICANS (17, 27, 37, 38). Van de Donk and colleagues found that the use of tocilizumab before teclistamab treatment appeared to reduce the incidence of CRS without new safety events or an impact on the response to teclistamab (39). ICANS, associated with a cytokine storm that allows high concentrations of cytokines to transit into the cerebrospinal fluid, usually occurred concurrently with or following CRS (40, 41). In this analysis, ICANS ranged from 2% to 11% at any grade and 0% to 2% at grade ≥ 3. However, Costa and colleagues reported that one patient treated with alnuctamab died in the study in the setting of CRS, with a potential infection as a contributing factor (21). In response to the serious concern of infections associated with BsAbs, a consensus recommendation from a panel of 13 global experts focused on infection monitoring, prophylaxis and treatment for patients with MM (42). Our analysis showed that any grade and grade ≥ 3 infections occurred in 48% (95%CI: 37%-59%) and 22% (95%CI: 19%-24%) of patients treated with BsAbs. The common infections were Covid-19, pneumonia and upper respiratory tract infection. Moreau and colleagues reported 12 deaths from Covid-19, and Lesokhin and colleagues reported 3 deaths from septic shock (18, 28). The infection risk factors in MM patients treated with BsAbs vary, such as dysfunction of the adaptive immune response, neutropenia, and the use of immunosuppressive agents (43–45). Future BsAb trials should take the incorporation of various prophylactic measures into consideration to prevent serious or even fatal infections. In addition, there are some similarities in the guidelines of BsAbs and CAR T-cell therapies, and a part of infection-related lessons can be drawn from CAR T-cell therapy (42, 46).

This study also presented significantly lower risks of neutropenia and lymphopenia among patients treated with non-BMCA-targeted BsAbs compared to those treated with BMCA-targeted BsAbs. Farah and colleagues thought that this was the result of the nuclear factor κB and c-Jun N-terminal kinase activation, which was caused by BCMA overexpression, and played a role in T-cell proliferation and cytokine release to increase the production of neutrophils (47, 48). The inhibition of BCMA expression led to a decrease in lymphocyte proliferation and neutrophil production. Moreover, a significant difference in the incidence of fatigue and pyrexia was observed between the two groups, which may be related to non-specific factors, such as disease characteristics and patient factors. Even without inclusion, skin-related events were observed in GPRC5D-targeted BsAbs therapy, with the most common events being exfoliation, pruritus, and dry skin. Despite their frequency, these events were primarily low-grade and responsive to both oral and topical glucocorticoid treatment (33).

While this study adhered to stringent selection and exclusion criteria, several limitations warrant consideration. Firstly, all enrolled studies were single-arm trials. Secondly, different targets (GPRC5D, FcRH5 and CD38) were classified as non-BCMA targets, and lastly, the drugs used varied among the studies. All of the above may cause bias. Although this study did not fulfill the above features completely, overall, the bias risk of study quality was acceptable.

In conclusion, BsAbs emerge as a promising therapeutic class for RRMM. This analysis indicated that opting for non-BCMA-targeted BsAbs therapy may present a more favorable treatment response and enhanced tolerability. On the contrary, BCMA-targeted BsAbs therapy appears to be associated with a heightened risk of ICANS. Our findings underscored the importance of carefully considering the choice of BsAbs therapy in clinical applications, with potential implications for optimizing patient outcomes and safety.

## Data availability statement

The original contributions presented in the study are included in the article/**Supplementary Material**. Further inquiries can be directed to the corresponding author.

## Author contributions

XW: Conceptualization, Data curation, Formal analysis, Investigation, Methodology, Writing – original draft. AZ: Conceptualization, Writing – review & editing. JZ: Writing – review & editing. TN: Funding acquisition, Writing – review & editing.
