# Supplementary material for: Efficacy and safety of bispecific antibodies therapy for relapsed or refractory multiple myeloma: a systematic review and meta-analysis of prospective clinical trials
Source: Front Immunol. 2024 Feb 28;15:1348955. doi: 10.3389/fimmu.2024.1348955 (PMC10933024; doi:10.3389/fimmu.2024.1348955)
Supplement: Supplementary file 1 [file DataSheet_1.docx]

Table S1. Modified MINORS evaluation of included studies

| **Reference** | **Study aim** | **Consecutive patients** | **Data collection methodology** | **Reported endpoints** | **Outcome evaluation bias** | **Equivalent groups** | **Statistical methods** | **Follow-up period** | **Follow-up loss** | **Overall scores** |
| --- | --- | --- | --- | --- | --- | --- | --- | --- | --- | --- |
| Moreau et al., 2022^27^ | 2 | 2 | 2 | 2 | 2 | 0 | 2 | 1 | 0 | 13 |
| Touzeau et al., 2022^19^ | 2 | 2 | 2 | 2 | 2 | 0 | 2 | 1 | 0 | 13 |
| Sun et al., 2023^25^ | 2 | 2 | 2 | 1 | 1 | 0 | 2 | 1 | 0 | 11 |
| Lee et al., 2023^22^ | 2 | 2 | 2 | 2 | 0 | 0 | 1 | 1 | 0 | 10 |
| Lesokhin et al., 2023^17^ | 2 | 2 | 2 | 2 | 2 | 0 | 2 | 1 | 2 | 15 |
| Voorhees et al., 2022^14^ | 2 | 2 | 2 | 2 | 1 | 0 | 2 | 1 | 0 | 12 |
| Wong et al., 2022^15^ | 2 | 2 | 2 | 2 | 0 | 0 | 2 | 1 | 0 | 11 |
| Costa et al., 2019^20^ | 2 | 2 | 2 | 2 | 1 | 0 | 0 | 1 | 0 | 10 |
| Raab et al., 2023^24^ | 2 | 2 | 2 | 2 | 1 | 0 | 2 | 1 | 0 | 12 |
| Schinke et al., 2023^23^ | 2 | 2 | 2 | 1 | 1 | 0 | 2 | 1 | 0 | 11 |
| Carlo-Stella et al., 2022^26^ | 2 | 2 | 2 | 1 | 0 | 0 | 2 | 1 | 0 | 10 |
| Lesokhin et al., 2022^18^ | 2 | 2 | 2 | 1 | 0 | 0 | 2 | 1 | 0 | 10 |
| Trudel et al., 2021^16^ | 2 | 2 | 2 | 1 | 0 | 0 | 2 | 1 | 0 | 10 |
| Mohan et al., 2022^21^ | 2 | 2 | 2 | 1 | 0 | 0 | 1 | 1 | 0 | 9 |
